# Sir Astley Cooper’s Bath

**Published:** 1854-04

**Authors:** 


### SIR ASTLEY COOPER'S BATH.

Sir Astley Cooper was the most eminent surgeon of his time, and he lived to a good old age, and although he wore silk stockings in the depth of an English winter, he seldom took cold, which exemption he attributed mainly to his morning bath, which he describes as follows :

" Immediately on rising from bed, and having all previously ready, take off your night dress, then take up from your earthen pan of two gallons of water a towel, quite wet but not dropping; begin at your head, rubbing hair and face, and neck and ears well; then wrap yourself behind and before, from neck to chest, your arms, and every portion of your body. Remand your towel into the pan, charge it afresh with water, and repeat once all I have mentioned, excepting the head, unless that be in a heated state, when you may do so, and with advantage. Three minutes will now have elapsed. Throw your towel into the pan, and then proceed with two coarse long towels, to scrub your head, and face, and body, front and rear, when four minutes will have you in a glow ; then wash and hard rub your feet, brush your hair, and complete your toilette; and trust me that this will give new zest to your existence. A mile of walking may be added with advantage."

Women and those who are delicate, and who are easily chilled, may modify Sir Astley's mode by adopting that which is described in the following language of a lady to a lady :

### A LADY'S BATH.

" You only want a basin of water, a towel, a rag, and five minutes time. When you get up in the morning pin a petticoat very loosely at the waist, draw your arms out of the sleeves of your chemise, and let it drop to your waist. Take your rag, well wetted, and slap your head and shoulders, rub your arms and chest, and throw handfuls of water around your ears and back of the neck. Then throw your towel across your back and 'saw' it dry. Rub fast until you are quite dry. Put on your chemise sleeves, draw on a night gown, to keep from chilling, while you tuck your skirts up under one arm, until you wash and dry one limb ; drop that side and do the other likewise, and be sure that the small of the back and sides get their full share of rubbing. This done, sit down, dip one foot in the basin, rub and dry it, put on your stocking and shoe, and then wash the other."

When needed, I am in the habit of advising the following, which, as a general rule, I think preferable to all others, because it is easily performed, costs nothing, and is practicable wherever there is a rag and a pint of cold water ; it leaves no ground of excuse for not performing it, and consequently there is no obstacle to its general employment.

It is my opinion, founded on observation, that a daily bath, to one in good health, is not only not beneficial, but is injurious, while it deprives a man of a valuable prophylactic when he is really sick. A MAN WHO IS WELL, SHOULD LET HIMSELF ALONE! I know very well there is a kind of furor in certain quarters about cold baths, and shower baths. It is often described as a delightful operation, and its healthfulness painted in glowing language. But is it true? It is wonderful how a community will sometimes take up a plausible idea, and run away with it, never stopping to investigate its propriety, its truthfulness, or its safety.

A daily bath, shower or otherwise, is a modern invention, devised to sell bath-tubs. I personally have known but two men, who acknowledged to a daily shower bath, literally a shower-bath every day. One of them died years ago of chronic diarrhœa, the other was a hydropathist, a great stout raw-boned six footer. I sat at the same table with him for many months; he was always bathing, and was always sick, he would frequently souse himself in cold water head and ears, two or three times a day. Does any reader of mine know any old man who has been a daily cold water bather all his days, or even for any five years of his life? Did Preisnitz, who gloried in cold water, live to be an old man? Does the observant reader know any man, dead or alive, who practiced a daily cold water bath for three consecutive years, and who enjoyed any remarkable good health, and who did not have good health before he began? Have we any written record of any nation, whose inhabitants practised as a general thing daily cold water bathings? These are inquiries which every reflecting man ought to make, and when they are answered, to conduct himself accordingly.

When a man is not well, bathing of some kind is advisable under certain circumstances, but it should not be continued too long; as soon as he is well he ought to stop. Once or twice a week persons may advantageously perform the following, if in good health, for the sake of personal cleanliness; if ailing, oftener:

### TOWEL BATH.

As a general rule, the best method is to dip a coarse cloth or a coarse linen or tow or hempen glove in cold water, squeeze it so that the water shall not dribble about, lay it flat on the hand, and with breast projecting and mouth closed, begin over the breast, on getting out of bed in the morning, and rub fast and hard, gradually extending it all over the body, as far as you can reach in every direction. This operation should be performed within ten minutes in summer, and within three or four in winter. Keep on the stockings, and when done, dress quickly, and go to the fire, if in cool weather, or take some exercise, active enough to make you feel comfortably warm.

